# Multiferroic Pb(Zr_0.52_Ti_0.48_)O_3_-CoFe_2_O_4_ Janus-Type Nanofibers and Their Nanoscale Magnetoelectric Coupling

**DOI:** 10.3390/nano16010002

**Published:** 2025-12-19

**Authors:** Qingfeng Zhu, Ting Wang, Junfeng Zhao, Haijuan Mei, Weiping Gong

**Affiliations:** Guangdong Provincial Key Laboratory of Electronic Functional Materials and Devices, Huizhou University, Huizhou 516007, China; qfzhu@hzu.edu.cn (Q.Z.);

**Keywords:** multiferroic nanofibers, Janus-type, magnetoelectric coupling

## Abstract

One-dimensional (1D) multiferroic composite nanofibers are known to exhibit enhanced magnetoelectric (ME) coupling compared to their thin-film and bulk counterparts with similar compositions. While measuring their local ME coupling at the nanoscale is essential for understanding multiferroic interactions, it remains challenging due to their complex structure. In this work, multiferroic Pb(Zr_0.52_Ti_0.48_)O_3_-CoFe_2_O_4_ (PZT-CFO) Janus-type nanofibers were synthesized by electrospinning. This unique structure is expected to provide a more compact and continuous interface between the ferroelectric and ferromagnetic phases compared to core–shell configurations. X-ray diffraction confirmed the coexistence of the perovskite PZT and spinel CFO phases without detectable impurities. The Janus configuration was directly verified by scanning electron microscopy and Kelvin probe force microscopy, which revealed a distinct surface potential contrast between the two halves of a single nanofiber. Magnetic hysteresis loops demonstrated the macroscopic ferromagnetic behavior of the nanofiber assembly. Local magnetoelectric coupling was probed using piezoresponse force microscopy under an applied magnetic field. An enhancement of the intrinsic piezoresponse from 15 pm to 19 pm. was observed upon applying an 8000 Oe magnetic field, providing direct evidence of strain-mediated ME coupling at the nanoscale. Although no ferroelectric domain switching was observed, likely due to the substrate clamping effect, the observed piezoresponse modulation confirms the functional ME interaction. These findings suggest that the Janus nanofibers hold promise for applications in one-dimensional multiferroic devices.

## 1. Introduction

Multiferroic materials have garnered significant attention from researchers in recent years due to their simultaneous possession of ferroelectric, ferromagnetic, and ferroelastic orderings, as well as their unique magnetoelectric (ME) coupling effect [1,2,3]. The ME coupling effect, which refers to the ability to control magnetization via an electric field or polarization via a magnetic field, holds not only great scientific significance but also broad application prospects [4,5]. It has the potential to bring revolutionary advances to industries such as actuators, sensors, memory devices, and microelectronics. However, single-phase multiferroic materials typically exhibit very weak ME coupling at room temperature, hindering their practical application [6,7]. Consequently, research focus has gradually shifted towards multiferroic composite materials. These composites are typically heterostructures formed by combining ferroelectric and ferromagnetic materials. Their ME coupling is mediated by mechanical stress transfer at the interfaces, utilizing the mutual coupling between the piezoelectric effect of the ferroelectric material and the magnetostrictive effect of the ferromagnetic material [8,9,10,11]. Therefore, to achieve a large ME effect, in addition to selecting component phases with strong intrinsic effects, a high-quality interfacial connection between the two phases is essential [12]. After more than a decade of effort, significant progress has been made in the research of multiferroic composites [13,14]. Notably, substantial ME coupling effects at room temperature have been realized in composite systems [15,16,17,18,19], and a series of prototype devices based on the ME effect have been developed [5,20,21].

Nanostructured composite multiferroics have attracted extensive research interest due to their rich interface and size effects, as well as their potential for promoting the multi-functionality, integration, and miniaturization of devices [22,23,24]. As we all know, suspended nanofibers can substantially reduce substrate constraints and possess a high aspect ratio, which can amplify the displacement induced by either the piezoelectric or magnetostrictive effect, thereby significantly enhancing the ME coupling of the material. When compared to thin-film geometries of the same material composition, composite nanofibers exhibit a magnetoelectric (ME) output that is often enhanced by several orders of magnitude [25]. This is because the one-dimensional form factor mitigates the restrictive “clamping” effect typically imposed by a substrate, thereby maximizing the product of the magneto-piezo coupling. This theoretical prediction has been validated in prior studies on core–shell nanofibers. Notable examples include CoFe_2_O_4_-Pb(Zr_0.52_Ti_0.48_)O_3_ (CFO-PZT) [26] and CoFe_2_O_4_-BiFeO_3_ (CFO-BFO) [27] systems. Currently, the primary structures for multiferroic composite nanofibers are randomly mixed, core–shell, and side-by-side (Janus) configurations [28]. Among these three morphologies, core–shell and randomly mixed composite nanofibers are the easiest to fabricate. The randomly mixed structure offers a large net contact area and is easily produced via electrospinning, but it suffers from significant interdiffusion between the two phases [29]. Core–shell nanofibers also benefit from a large contact area but often face the issue of inadequate interfacial bonding due to the non-uniform contraction of ceramic fibers [30]. In this context, the quest for an optimal microstructure that maximizes interfacial coupling while minimizing fabrication challenges remains a key research focus. The Janus structure, where both phases are joined at an interface that runs longitudinally along the length of a nanofiber, maintains a reasonably large contact area while ensuring a tight interfacial connection [31], making it a promising candidate for achieving stronger ME coupling effects.

Electrospinning has been widely adopted for synthesizing composite polymer [32,33,34] and ceramic nanofibers [35,36,37] due to its operational convenience and versatility. Previous work successfully prepared BaTiO_3_-CoFe_2_O_4_ (BTO-CFO) Janus nanofibers via electrospinning and confirmed their multiferroicity [28]. However, the local ME coupling effect has not been effectively measured due to the characterization challenges posed by their complex structure. Recently, researchers successfully synthesized aggregates of Janus nanofibers and detected the ME coupling of the aggregates using second harmonic generation (SHG) polarimetry under different magnetic field orientations [38]. Nevertheless, the lateral resolution of SHG measurements is limited by the laser spot size, making it difficult to characterize ME coupling at the nanoscale. To the best of our knowledge, ME coupling measurement on a single multiferroic Janus nanofiber has not been reported. In this paper, Pb(Zr_0.52_Ti_0.48_)O_3_-CoFe_2_O_4_ (PZT-CFO) Janus-type nanofibers were synthesized via electrospinning and their morphology, crystalline structure, and magnetoelectric coupling properties were systematically investigated.

## 2. Experimental Details

### 2.1. Synthesis of PZT-CFO Single-Phase and Janus Nanofibers

PZT nanofibers: PZT nanofibers were synthesized via electrospinning using procedures based on previously described work [39]. Briefly, tetrabutyl titanate and zirconium nitrate were dissolved in 2-methoxyethanol solution. Simultaneously, lead acetate was dissolved in glacial acetic acid and then the solution was added dropwise into the mixed zirconium-titanium salt solution while stirring continuously to form a light yellow, transparent precursor solution. An appropriate amount of solvent with the volume ratio of ethylene glycol monomethyl to acetic acid at 5:1.3 was added to the precursor solution, and the concentration of the final mixed solution was adjusted to 0.4 mol/L. A homogeneous spinning solution was prepared by incorporating polyvinylpyrrolidone (PVP, MW = 1,300,000) into the precursor solution, achieving a concentration of 0.06 g/mL. The solution was subsequently stirred for 4 to 6 h to ensure complete homogenization.

The precursor solution was electrospun at a flow rate of 0.3 mL/h (syringe pump, NE-500, New Era Pump SystemsInc., Farmingdale, NY, USA) under an applied voltage of 20 kV. The collector, consisting of Pt/Ti/SiO_2_/Si substrates, was positioned 15 cm from the needle tip. Following deposition, the collected fibers were subjected to a series of heat treatments: initial drying at 120 °C for 4 h, subsequent heating at 450 °C for 1 h, and final annealing at 750 °C for 2 h in air.

CFO nanofibers: CFO nanofibers were also synthesized using electrospinning according to previously outlined processes [39]. Here, Here, cobalt nitrate, iron nitrate, and citric acid were dissolved in a mixed solution of ethanol and distilled water with a volume ratio of 1:1 under continuous stirring. To facilitate fiber formation, polyvinylpyrrolidone (PVP) with a molecular weight of 1.3 million was dissolved in the precursor solution. The solution was stirred for 4–6 h, maintaining the polymer concentration at 0.06 g/mL. CFO nanofibers were electrospun, dried, and calcined as described for PZT nanofibers.

**Figure 1:**
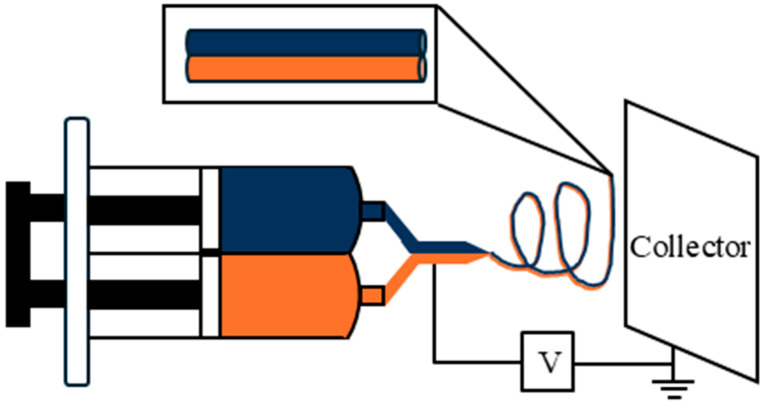
The schematic setup of the electrospinning system.

Janus nanofibers: The PZT and CFO precursor solutions were co-electrospun simultaneously through a customized dual-needle spinneret. The core setup, illustrated schematically in Figure 1, comprised three essential components: a high-voltage power supply, a grounded collector, and two syringe pumps. To guarantee a consistent and equal flow rate for both solutions, the two separate syringes were actuated by a single pump controller to ensure flow rate synchronization. Electrospinning, drying, and calcination were performed as described above for the individual PZT and CFO nanofibers.

### 2.2. Characterization

The crystalline phases of the nanofibers were characterized by X-ray diffraction (XRD, Rigaku MiniFlex 600). Morphological analysis was performed using scanning electron microscopy (SEM, Hitachi S4800). An atomic force microscope (AFM, Asylum Research MFP-3D) was employed to investigate the local electrical and electromechanical properties; specifically, its Kelvin probe force microscopy (KPFM) module was used for surface potential mapping, while the ferroelectric and magnetoelectric coupling characteristics were examined via piezoresponse force microscopy (PFM) with a variable field module (VFM), following a procedure detailed in our previous work [27]. The macroscopic magnetic behavior was measured with a vibrating sample magnetometer (VSM, Yingpu VSM-175).

## 3. Results and Discussion

### 3.1. Crystalline Structure

**Figure 2:**
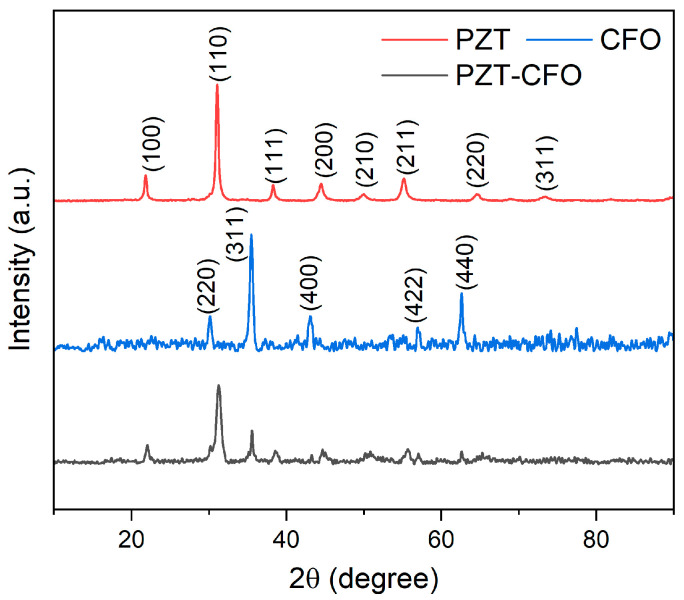
XRD pattern of PZT, CFO and PZT-CFO nanofibers.

Figure 2 shows the X-ray diffraction (XRD) patterns of the pure PZT, pure CFO, and the PZT-CFO Janus composite nanofibers. For the composite sample, all the diffraction peaks can be well-indexed to the perovskite phase of PZT (JCPDS no. 33-0784) and the spinel phase of CFO (JCPDS no. 22-1086). No additional impurity peaks are detected, indicating the successful synthesis of a biphasic composite without unwanted chemical reactions during the high-temperature calcination process. To provide a more comprehensive and quantitative characterization, we performed further analysis on the XRD data. First, the relative weight fraction of the PZT and CFO phases was estimated using the reference intensity ratio (RIR) method. This involved integrating the areas of the main characteristic diffraction peaks—the PZT (101) peak and the CFO (311) peak—and normalizing them with their respective RIR values. The calculation revealed that the weight fraction of the PZT phase was approximately 55 wt% and that of the CFO phase was 45 wt%. It should be noted that this is a semi-quantitative estimation, and the result may be influenced by factors such as preferred orientation. Furthermore, the significant broadening of the diffraction peaks provides information about the crystallite size. The CFO peaks are visibly broader and less intense than those of the PZT and PZT-CFO composite nanofibers, suggesting a smaller crystallite size and lower crystallinity for the CFO component within the composite. To quantify this, the average crystallite size for both phases was calculated using the Scherrer equation, D = Kλ/(βcos θ), where K is the shape factor (taken as 0.9), λ is the X-ray wavelength (0.15406 nm for Cu Kα), β is the full width at half maximum (FWHM) after instrument broadening correction, and θ is the Bragg angle. The calculations were performed on the PZT (101) and CFO (311) peaks. The results show that the average crystallite size of the PZT phase is approximately 35 nm, whereas the CFO phase possesses a smaller average crystallite size of 20 nm. These quantitative results are in excellent agreement with the visual observation of the XRD patterns, confirming the lower crystallinity of the CFO phase.

### 3.2. Morphology and Surface Potential

**Figure 3:**
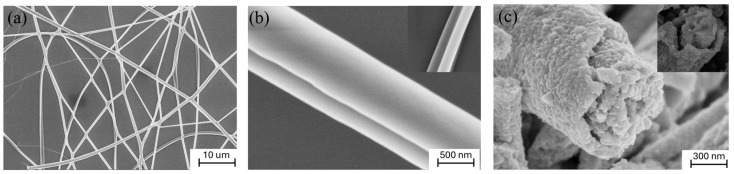
Morphology and Janus structure of the PZT-CFO nanofibers. (**a**) A low-magnification SEM image showing the continuous and uniform morphology of the fibers; (**b**) High-magnification SEM images revealing the distinct side-by-side structure of a single Janus nanofiber; (**c**) SEM images of core–shell CFO-BFO nanofiber [27].

Representative SEM images of the synthesized PZT-CFO Janus nanofibers are presented in Figure 3. As shown in Figure 3a, the nanofibers are continuous and uniform with an average diameter of approximately 1 µm, exhibiting a markedly smoother surface than the BFO-CFO core–shell fibers reported in our previous work. This enhanced smoothness is attributed to the finer grain size of the PZT phase. A higher-magnification image in Figure 3b clearly reveals the characteristic side-by-side (Janus) topology of an individual nanofiber. A direct comparison with our previously reported core–shell fibers (Figure 3c) highlights a distinct morphological advantage of the Janus architecture: the interface between the PZT and CFO phases in the Janus structure is more direct and intimate, exhibiting tighter interfacial bonding with minimal gaps. This enhanced contact is crucial for efficient strain transfer, which is fundamental to achieving strong magnetoelectric coupling.

**Figure 4:**
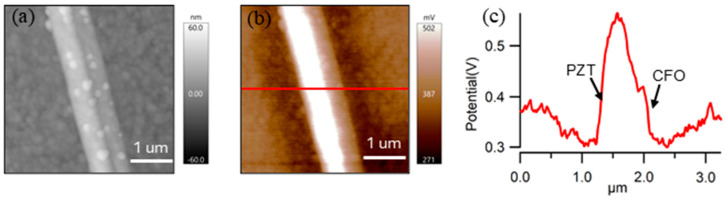
Nanoscale surface potential analysis of a single PZT-CFO Janus nanofiber. (**a**) AFM topography image; (**b**) Corresponding surface potential (CPD) map, showing a pronounced contrast between the two halves of the fiber; (**c**) Line profile of the surface potential extracted along the solid line in (**b**), quantitatively illustrating the lateral heterogeneity.

The Janus-type structure of the nanofiber is further confirmed by Kelvin probe force microscopy. The AFM topography and corresponding surface potential distribution of a typical PZT-CFO Janus nanofiber are presented in Figure 4. As shown in Figure 4a, the total diameter of the Janus nanofiber is approximately 1 µm, consistent with SEM observations. The surface potential mapping and a corresponding line profile are displayed in Figure 4b and Figure 4c, respectively. A clear surface potential contrast is observed between the nanofiber and the silicon substrate. Notably, the left region of the nanofiber exhibits a significantly higher surface potential than the right region. We attribute this pronounced difference to the distinct material phases constituting the Janus structure: the left region is likely the PZT phase, while the right corresponds to the CFO phase. This assignment is strongly supported by the ferroelectric nature of PZT, whose remanent polarization induces bound surface charges, attracting a substantial amount of screening charges from the conductive Si substrate and the ambient environment, thereby elevating the local contact potential difference. In addition to the lateral heterogeneity, a noticeable potential sharpening is observed along the edges of the nanofiber. This edge effect can be attributed to a combination of geometric electric field concentration, which enhances the local density of unscreened polarization charges, and the inherent tip-sample convolution artifact in KPFM, where the probe interacts simultaneously with the fiber’s top, side, and the adjacent substrate at the edges. Collectively, the distinct surface potential between the two halves of the fiber, complemented by the characteristic edge potential profile, provides compelling evidence for the successful formation of a Janus heterostructure with phase-specific electronic properties.

### 3.3. Ferromagnetic Property

**Figure 5:**
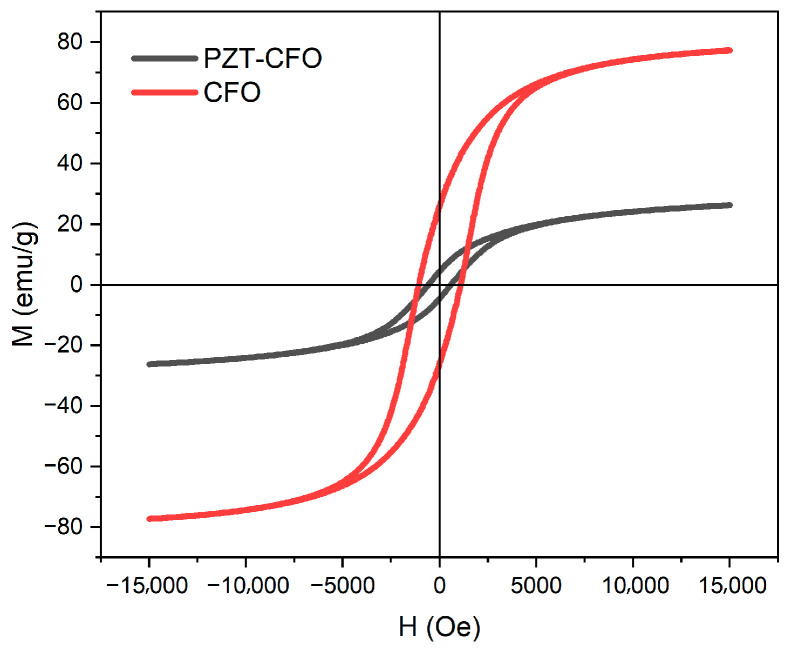
VSM magnetic hysteresis loops of PZT-CFO Janus and CFO nanofibers.

As demonstrated by the magnetic hysteresis loop in Figure 5 measured using a VSM, the PZT-CFO Janus nanofibers unambiguously exhibit room-temperature ferromagnetism. Quantitatively, the saturated magnetization Ms and remnant magnetization Mr of the Janus nanofibers are measured to be respectively 25.8 emu/g and 4.18 emu/g, with a corresponding coercive field of approximately 600 Oe. These key magnetic parameters are significantly lower than those of single-phase CFO nanofibers (Ms = 77.4 emu/g, Mr = 27.2 emu/g, Hc = 1100 Oe). This reduction in magnetic performance can be primarily attributed to two factors. Firstly, the most direct reason is the “dilution effect” [40]: In the Janus structure, the ferromagnetic CFO phase coexists with the non-ferromagnetic PZT phase. This reduces the proportion of magnetic material per unit mass of the sample, directly leading to a decrease in the overall saturation and remnant magnetization. Secondly, the interfacial effect between CFO and PZT also plays a crucial role [41]. At the interface, lattice mismatch may induce strain, or elemental interdiffusion could create a magnetically disordered “dead layer.” This would weaken the intrinsic magnetism of the CFO phase and affect the pinning of magnetic domain walls, ultimately resulting in a lower coercive field. Therefore, the observed magnetic properties of the PZT-CFO Janus nanofibers are a combined result of the intrinsic magnetism of the CFO phase and the unique Janus composite structure, governed by both dilution and interfacial effects. In addition to the dilution effect and interfacial effects, the smaller grain size and less developed crystallinity of the CFO phase within the Janus structure are also likely contributing factors to the observed decrease in coercive field.

### 3.4. Ferroelectric and ME Coupling Properties

**Figure 6:**
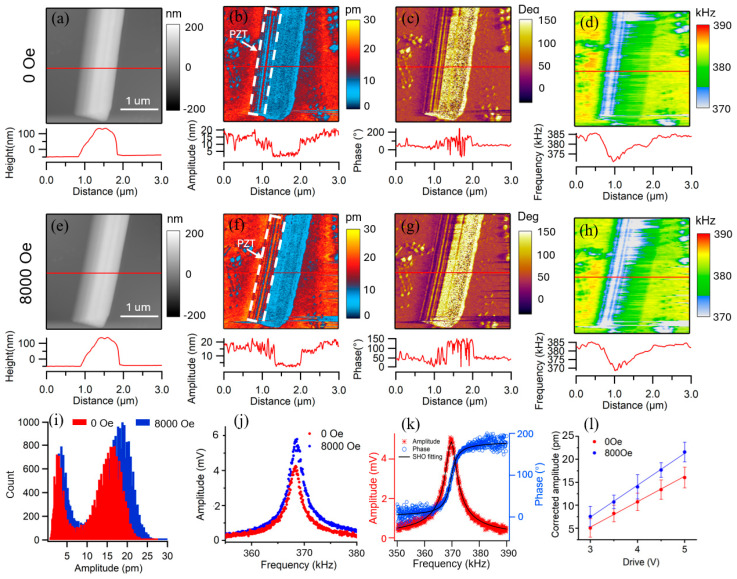
The mapping of vertical piezoresponse in the PZT-CFO Janus-type nanofibers before and after the application of external magnetic field by VFM: (**a**) nanofiber topography, (**b**) amplitude, (**c**) phase, (**d**) resonance frequency images with line scan across the fiber under 0 Oe; (**e**) nanofiber topography, (**f**) amplitude, (**g**) phase, (**h**) resonance frequency images with line scan across the fiber under 8000 Oe; (**i**) histogram distribution of PFM amplitude on PZT nanofiber (shown in the white box in (**b**,**f**)); (**j**) single-point piezoresponse measured on the PZT nanofiber with 0 Oe and 8000 Oe with an AC voltage of 4 V, (**k**) SHO model and fitting; (**l**) intrinsic piezoresponse versus the excitation voltage for 0 Oe and 8000 Oe.

The piezoelectric characteristics of the Janus nanofibers were quantified using dual-frequency resonance tracking (DFRT) piezoresponse force microscopy. This technique allows for the extraction of the intrinsic vertical piezoresponse by calibrating the quality factor (Q), as detailed in Figure 6. Subsequently, an in-plane magnetic field was applied via the variable field module (VFM) to probe magnetoelectric coupling. The resulting changes in the PFM amplitude and phase were monitored to reveal the strain-mediated interaction between the ferromagnetic and ferroelectric phases. The exceedingly diminutive nanofiber interface results in minimal ME coupling caused by the magnetic field, rendering measurement exceedingly challenging; nonetheless, our PFM-based technology provides a practical approach to validate such ME coupling at the nanoscale. The diameter of the Janus nanofiber for PFM test is around 1 µm, while its height is a little more than 100 nm, as revealed by the topographic image in Figure 6a. To acquire the PFM amplitude images before and after magnetic field application (Figure 6b,f), the local piezoelectric response of the fiber was measured. This was achieved by monitoring the resultant electromechanical oscillation generated from an AC electric field applied through the conductive tip. It is seen from Figure 6b that the amplitude on the left side of the fiber is significantly larger than that on the right side, indicating that the piezoelectric response on the left side of the fiber is significantly larger than that on the right side. Thus, it can be determined that the left side of the fiber is a PZT phase with piezoresponse, while the right side is a CFO phase with no piezoresponse. Within the PZT phase, the PFM amplitude exhibits lateral variations, likely influenced by underlying topography. Nevertheless, the application of an 8000 Oe in-plane magnetic field induces a clear modification in the amplitude signal, as evidenced by the data in Figure 6f. Comparison of the histogram distribution of PFM amplitude on PZT nanofiber (shown in the white box in Figure 6b,f) between 0 and 8000 Oe is shown in Figure 6i, indicating a clear shift toward higher piezoresponse from 15 pm to 19 pm under the in-plane magnetic field. This enhancement originates from a strain-mediated mechanism, whereby magnetostrictive strain generated in the CFO phase is elastically transferred to the PZT phase, inducing an additional piezoelectric response. No oblivious domain structure is observed in the phase image, as shown in Figure 6c, which is probably due to the polycrystalline nanostructures of the PZT nanofibers. The phase contrast of the fiber in Figure 6g does not change significantly after applying the magnetic field, indicating that the domain does not switch after applying the magnetic field. The lack of domain switching is attributed to a clamping effect, primarily from the rigid substrate to which the entire Janus fiber is attached, which restricts the overall strain transfer and prevents the collective reorientation of domains. Additionally, the mechanical constraint exerted by the adjacent CFO phase may also play a secondary role. From the resonance frequency images shown in Figure 6d,h, it is observed that the resonance frequency on the left side of the fiber is much lower than that on the right side and the substrate, indicating the left side of the fiber has a lower elastic modulus, since the resonance frequency measured by PFM is proportional to the elastic modulus of the material. It is reported in our previous work that elastic modulus of PZT fiber is about 45 GPa, while the elastic modulus of CFO fiber is about 150 GPa [36], which further confirms that the left side of the fiber is PZT phase and the right side is CFO phase.

To circumvent topographical interference in PFM measurements, single-point tests were conducted on the PZT nanofiber. The piezoresponse was quantified at identical locations before and after magnetic field application (Figure 6j), confirming a definitive increase. To further substantiate this enhancement, the intrinsic PFM response was statistically analyzed by averaging data from nine randomly selected points. The deflection response was fitted to a simple harmonic oscillator (SHO) model (Figure 6k) to extract the voltage-dependent piezoresponse. As plotted in Figure 6l, the PZT nanofiber exhibits the expected linear piezoelectric behavior. The effective piezoelectric coefficient d_33_*, derived from the slope of the piezoresponse vs. voltage plot (Figure 6l), increased from 4.91 ± 0.26 pm/V at 0 Oe to 6.75 ± 0.29 pm/V at 8000 Oe, unambiguously establishing the magnetic field-induced enhancement of the piezoresponse. The increase in effective piezoelectric coefficient may originate from the magnetostrictive stress of the CFO phase under a magnetic field, which assists in aligning the ferroelectric domains along the measurement axis.

## 4. Conclusions

In summary, multiferroic Pb(Zr_0.52_Ti_0.48_)O_3_-CoFe_2_O_4_ (PZT-CFO) Janus-type nanofibers were successfully synthesized via a customized electrospinning technique. A comprehensive suite of characterization techniques was employed to investigate their properties:

• Structural and morphological analysis through X-ray diffraction (XRD), scanning electron microscopy (SEM), and atomic force microscopy (AFM) confirmed the biphasic composition, high phase purity, and the distinctive side-by-side architecture of the nanofibers.
• Nanoscale functional mapping using Kelvin probe force microscopy (KPFM) revealed a pronounced surface potential contrast between the two halves of an individual fiber, providing direct evidence for the Janus heterostructure with phase-specific electronic properties.
• Macroscopic magnetic measurement by vibrating sample magnetometer (VSM) demonstrated clear room-temperature ferromagnetism, with magnetic parameters influenced by both dilution and interfacial effects within the composite.
• Local electromechanical and magnetoelectric characterization was achieved via dual-frequency resonance tracking piezoresponse force microscopy (DFRT-PFM). This critical analysis quantified the intrinsic piezoelectric response and, under an applied magnetic field, captured a definitive enhancement from 15 pm to 19 pm.

The central finding of this work is the direct observation of magnetoelectric coupling at the nanoscale on a single Janus nanofiber, mediated by strain transfer across the intimate interface. Although the substrate clamping effect suppressed full ferroelectric domain switching, the unambiguous modulation of the piezoresponse by a magnetic field validates the functional ME interaction. This study not only establishes a viable pathway for creating one-dimensional multiferroic nanomaterials but also demonstrates the efficacy of advanced PFM techniques for probing local magnetoelectric phenomena, paving the way for their potential applications in miniaturized sensors, memory devices, and spintronics.

## Data Availability

The original contributions presented in this study are included in the article. Further inquiries can be directed to the corresponding authors.
